# More rumination and less effective emotion regulation in previously depressed women with preserved executive functions

**DOI:** 10.1186/s12888-014-0334-4

**Published:** 2014-11-27

**Authors:** Martin Aker, Catherine Harmer, Nils Inge Landrø

**Affiliations:** Clinical Neuroscience Research Group, Department of Psychology, Psykologisk institutt, University of Oslo, PO box 1094, Blindern, Oslo, 0317 Norway; Department of Psychiatry, Warneford Hospital, University of Oxford, Oxford, OX3 7JX UK

**Keywords:** Executive function, Emotion regulation, Rumination, Depression, Remitted MDD

## Abstract

**Background:**

Major depressive disorder is associated with very high recurrence rates, and specific vulnerability factors that increase the risk for repeated episodes should be identified. Impaired executive functions have repeatedly been found in remitted populations. The current study included both neutral and emotional executive tasks, and we expected to find impaired performance in unmedicated previously depressed women compared to controls. Furthermore, we hypothesized that the executive functions inhibition and shifting would be related to the ability to apply cognitive reappraisal and to avoid unhealthy rumination.

**Methods:**

Inhibition and shifting data derived from neutral and emotional computerized tasks, and questionnaire data on emotion regulation and trait rumination, were obtained from previously depressed (n = 109) and never-depressed women (n = 64) and analyzed in independent samples t-tests. A logistic regression analysis investigated the ability of emotion regulation and rumination to predict depression vulnerability. The associations of executive functions to emotion regulation and rumination were investigated in a series of linear regression analyses. Participants on psychotropic medication were excluded from all analyses of executive performance.

**Results:**

Previously depressed participants, the majority of which had experienced recurrent episodes, matched control participants on both neutral and emotional executive tasks. However, significantly more rumination and expressive suppression, and less cognitive reappraisal, were found in the previously depressed group. Executive function was unrelated to rumination and emotion regulation in this sample.

**Conclusions:**

Previously depressed women whose executive function was intact were characterized by ruminative tendencies and more frequent use of expressive suppression. Trait rumination and expressive suppression are known to increase depression risk, but were unrelated to executive functions in this population. This indicates that unhealthy emotion regulation strategies may be targeted directly in preventive interventions.

## Background

Depression is characterized by emotional and cognitive impairments including depressed mood, feelings of worthlessness, and diminished ability to think or concentrate [1]. Of the 15–20% who experience depression during their lifetime [2], 65–75% experience recurrent episodes [3,4]. The National Institute for Health and Clinical Excellence [5] has identified secondary prevention as a key goal in the long-term management of depression. High recurrence rates suggest that specific vulnerability factors increase the risk for developing repeated episodes of the disorder and these factors should be identified. One way to achieve this goal is to compare previously depressed subjects in remission, having a known vulnerability, and never depressed subjects, on relevant cognitive and emotional function dimensions.

Meta-analyses confirm that ongoing unipolar depression is reliably associated with impairments in multiple aspects of executive function [6,7]. Executive functions (EF) also tend to be impaired in participants with remitted Major Depressive Disorder (MDD), and the largest impairments are found in inhibitory control [8,9].

Emotional (or “hot”) cognition is also affected in depression. In particular, biased attention and perception of negative stimuli in dysphoric and clinically depressed individuals has been reported [10-12]. Importantly, biased attention to negative information has been found in both currently depressed and remitted participants [13,14].

Variations in the use of emotion regulation strategies like cognitive reappraisal, expressive suppression, and rumination play an important role in depression [12,15,16]. Individuals who have experienced depression in the past have been found to employ dysfunctional strategies more frequently (i.e. rumination, catastrophizing), and employ functional strategies less frequently (i.e. putting into perspective) [17]. However, negative findings have also been reported [15,18].

Compared to men, women have nearly twice the lifetime prevalence of depression [19]. Women tend to ruminate more, and the gender difference in rumination precedes the gender difference in depression, indicating that rumination may contribute to the sex difference in depression [20,21]. In rumination studies both trait and experimentally induced rumination are associated with impaired inhibition and switching in depressed individuals [15,22-25].

It has been reported that depressed patients show a specific failure to inhibit negative information, whereas formerly depressed individuals exhibited impaired inhibition of negative as well as positive information [26]. In another study, executive control functions with emotionally valenced material, and clinical phenomena like emotion regulation and rumination, were investigated in a sample of previously depressed subjects compared to never depressed individuals [15]. In that study a substantial proportion of participants in the clinical groups were taking medications. This is a compromising factor because antidepressant medication influences emotional processing and reduces the negative bias associated with depression [27]. The two aforementioned studies [15,26] used a negative affective priming task to assess inhibition, but negative priming is a controversial paradigm which has been criticized for low reliability [28]. It is weakly related to latent inhibition factors derived from other inhibition tasks, and researchers have questioned whether negative priming reflects active suppression of distracting information [29,30]. It is also a paradox that objectively better performance (faster reaction time to a probe) is interpreted as poorer inhibition, even though faster termination of the inhibitory effect would cause the same result. It was necessary to supplement the findings from J Joormann and IH Gotlib [15] and E Goeleven, R De Raedt, S Baert and EH Koster [26] with an investigation based on other measures of inhibition.

The aim of the current study was to investigate emotion regulation and executive control functions in unmedicated previously depressed and control participants. Inhibition was measured with the traditional Stroop paradigm and a modified version of the Stop-Signal task. Secondly, we also investigated the proposition that executive control in general, and in the processing of emotional material in particular, is related to depressive rumination and the emotion regulation strategy of cognitive reappraisal.

## Methods

### Ethics statement

The project was approved by the Norwegian Regional Committee for Medical and Health Research Ethics (REC South East D, 2011/2593/REK) and conducted in accordance with the Helsinki Declaration. All participants received both written and spoken information about the project, ethical and legal obligations of the researchers, and the rights of voluntary participants. Test administrators ensured that each participant had understood the information before informed consent was obtained by signature.

### Participant inclusion

We included female participants because, compared to men, women have nearly twice the lifetime prevalence of depression [19]. Two strategies were used for recruitment. Advertisements in local newspapers and online social media were used to recruit participants, with or without a history of depression, from the general local community. To ensure a sufficient number of participants with a history of depression, people discharged from a public mental health outpatient clinic were contacted and invited to participate. This outpatient clinic offers short-term treatment to patients that have a job and are at risk of long-term disability due to depression or anxiety. Inclusion criteria were female, age 18–65, no known neurological disorder, no history of severe head trauma, and good Norwegian language skills. Exclusion criteria were alcohol or drug abuse, current or previous manic episode or other psychotic disorder, current hypomanic episode, current depression.

### Clinical assessment and questionnaires

#### Diagnostic assessment

Diagnostic interviews were performed based on the MINI International Neuropsychiatric Interview 6.0. Some modules were omitted to reduce total time consumption and strain on the participants. The following modules were administered: major depressive episode, manic and hypomanic episodes, panic disorder, agoraphobia, social phobia, obsessive-compulsive disorder, posttraumatic stress disorder, psychotic disorders, anorexia, bulimia, and generalized anxiety disorder. All interviewers had received formal training in diagnostic assessment. With the exception of one case in which the participant declined, all interviews were audiotaped. The audiotaping allowed reviewing when the interviewer experienced any uncertainty about diagnostic decisions. Upon any uncertainty each interview was reviewed by author MA. After all inclusions were finalized, twenty-one cases which were still associated with some doubt were reviewed by a highly experienced clinical psychologist and researcher who was otherwise uninvolved with the project, and final diagnostic decisions were made by MA based on the expert recommendations.

#### Current symptoms

Symptom levels of depression and anxiety were measured using the Beck Depression Inventory II [31] and the Beck Anxiety Inventory [32].

#### Alcohol and drug use

Norwegian versions of the Alcohol Use Disorder Identification Test (AUDIT) [33] and Drug Use Disorder Identification Test (DUDIT) [34] were included to identify participants with alcohol or drug abuse. AUDIT and DUDIT are self-report forms consisting of 10 and 11 questions, respectively. Each question is accompanied by graded response alternatives and respondents are asked to check the alternatives that best represent their personal alcohol or drug habits and negative consequences of use.

#### Rumination

The Ruminative Responses Scale (RRS) is a 22 items self-report assessment of tendency to depressively ruminate. It comprises a three-factor structure that differentiates neutrally valenced and coping-oriented contemplation (reflection, five items) from passive and unproductive focus on problems and unachieved goals (brooding, five items) and depressive symptoms that are similar to BDI items (depression, 12 items) [35]. Respondents were instructed to use a 4-point scale to rate how often they react according to the 22 statements when feeling down, sad or depressed.

#### Emotion regulation

The Emotion Regulation Questionnaire (ERQ) was developed by JJ Gross and OP John [36]. It has ten items, each intended to measure one of the regulatory processes cognitive reappraisal or expressive suppression. Respondents were instructed to report how they control their emotions by responding to the ten statements on a 7-point scale, ranging from “strongly disagree” to “strongly agree”. The ERQ has six items for reappraisal and four items for suppression, explicitly referring to the regulation of positive as well as negative emotions.

### Cognitive measures

#### Non-emotional executive tasks

The Color-Word Interference Test (CWI) [37] is based on the procedure developed by J Stroop [38]. The CWI has four conditions, of which the first two assess baseline processing speed of color naming and reading. The third condition, Inhibition, is the traditional Stroop paradigm where colors are printed in letters with a different color than the word names, creating interference when the respondent is asked to report the printed color. The fourth condition, Inhibition/switching, has similar non-congruent color words, and requires the respondents to switch between reading and color naming.

#### The Emotional Picture Sorting Task (EPST)

The EPST was developed by authors MA and NIL [39] and is based on the same task structure as the Wisconsin Card Sorting Test (WCST) [40,41]. The WCST stimuli are comprised of cards depicting figures varying on three features: color, shape, and number. In the EPST, these stimuli have been changed to facial expressions with colored backgrounds, but the structure and procedure of this task was otherwise kept identical to the WCST. The total set of stimuli depicts four different individuals with four different emotional expressions for each individual. From the original WCST each *shape* was interchanged with one individual (i.e., triangle = individual 1, star = individual 2, cross = individual 3, circle = individual 4). *Number* on a WCST card was interchanged with facial expression (one = neutral expression, two = sad, three = happy, four = fearful). The colors red, green, yellow and blue were used as background colors in the pictures.

16 pictures, showing four adult female Caucasian individuals displaying four different expressions, were selected from the Karolinska Directed Emotional Faces database [42]. Graphic adjustments were performed in Adobe Photoshop CS5 software. Pictures were cropped using a 250*297 pixels frame. Eye level was kept center of the picture both horizontally and vertically. Brightness was increased, depending on the original picture, to make it appear similar for all pictures. Finally, background color was manipulated, producing four versions of each of the 16 pictures where the background color for each picture was red, green, yellow or blue. The final set of stimuli consists of 64 different pictures varying on the three dimensions color, person, and expression, and the set of 64 was used twice to allow a maximum of 128 trials. EPST was programmed in C++. See [39] for a more detailed description of this task.

#### Emotional Stop-Signal Task (ESST)

This task was based on the stop-signal paradigm of inhibitory control developed by GD Logan, WB Cowan and KA Davis [43]. The SST is a choice reaction time task where the subject makes a motor response by pressing a button corresponding to the direction (right or left) of a visually presented arrow. On a subset of trials, an auditory signal requires inhibition of the predominant motor response. In this emotional version of the SST, a picture of a human face displaying either a neutral or an angry expression was presented for 500 ms immediately before the target stimulus (arrow pointing right or left) on all trials. The test was administered in a blocked procedure with two practice blocks (40 + 20 trials) and four test blocks. The test blocks had 40 trials each, of which 25% were stop trials. Two blocks included only neutral faces (N) and two blocks included only angry faces (A). The sequence of the test blocks were either N-A-N-A or A-N-A-N, randomized between participants. The stop signal delay started on 250 ms and was adjusted in a tracking procedure to converge each participant’s performance on 50% successful stopping [44,45]. The main outcome variable was the Stop-Signal Reaction Time (SSRT) which is a time estimate of the inhibitory process. Separate SSRTs were calculated for neutral and angry blocks, based on the integration method [45]. Reaction time to go trials (GoRT) larger than 2.5 SD from mean for each participant were deleted before calculation of SSRT to reduce influence by extreme scores. GoRT and SSRT were found to be higher (i.e. slower) in angry compared to neutral blocks in a similar version of this task [46].

#### Intellectual functioning

The Wechsler Abbreviated Scale of Intelligence [47] subscales Vocabulary and Matrix reasoning served as indicators of general cognitive functioning.

### Procedure

In addition to the assessments described here, Verbal fluency and Digit span tests were administered, and participants provided cells for genetic sampling using buccal swabs. These data will not be presented in the current article. EPST and ESST were administered on a Dell Latitude D610 laptop computer with a 14.1” LCD screen using 1024 × 768 pixels at 32 bit color quality. External mouse and speakers were connected. All other tests were administered manually. After completion participants were compensated with an electronic debit card of 250 NOK (approximately €30). Total duration of the testing session, including MINI interview and questionnaires, was 100–120 minutes.

### Statistical analysis

All statistical analyses were performed in IBM SPSS 20. Two groups were defined a-priori based on the criterion history of depression following the research questions. Demographic and symptom characteristics, and all test outcome variables including executive functions and emotion regulation, were analyzed in independent samples *t*-tests between previously depressed and never depressed participants. A logistic regression analysis was performed using the ERQ variables, cognitive reappraisal and expressive suppression, and two RRS factors, brooding and reflection, as predictors of depression. A series of linear regression analyses were performed using executive functions variables as predictors of emotion regulation and rumination.

## Results

### Participant characteristics

201 women participated in the project. According to predefined exclusion criteria and based on information that was produced during the interview and testing session, data from 28 participants were excluded from all analyses due to the following reasons. Current or previous manic episode or other psychotic disorder (three participants), current hypomanic episode (two participants), current depression (17 participants). Three participants were excluded due to scores that were high and clearly deviant on AUDIT (one person, score 27) or on DUDIT (two persons, both scores 20). Three participants were excluded because their previous depressions were likely due to hormonal disturbances. These participants reported they had been formally diagnosed with hypothyreosis, they were untreated for hypothyreosis at the time of depression, and had not experienced depressive episodes while taking appropriate hormonal medication. Finally, data from 173 participants was available for analyses.

Twenty-nine currently present diagnoses, most of which were anxiety disorders, were registered in 24 of the remaining participants. These included agoraphobia (10), generalized anxiety disorder (7), bulimia (4), obsessive compulsive disorder (3), social phobia (2), posttraumatic stress disorder (2), and panic disorder (1). With one exception (posttraumatic stress disorder) all current diagnoses were found in previously depressed participants. Twenty-four participants reported using psychotropic medication, of which 18 used antidepressants (SSRI/SNRI), four used mood stabilizers, and two used other psychotropic medication (quetiapine, zopiclone). The participants using psychotropic medication were excluded from the analyses of executive function data because processing of emotional stimuli can be influenced by antidepressants [27] and perseverative behavior can be influenced by manipulation of serotonergic signaling in prefrontal brain areas [48]. Post-hoc analyses showed that these individuals did not perform significantly different from unmedicated participants.

### Between-groups comparisons: previously depressed vs. controls

#### Demographic and clinical characteristics

Descriptive information is presented in Table 1. Compared to the control group, the previously depressed participants reported more than double the symptom load as shown by BDI-II scores. They also reported significantly more anxiety symptoms. There were no differences between groups on age, education, general cognitive functioning, or alcohol and drug use.

**Table 1:** **Participant characteristics**

	**All** ^**a**^						**Without psychotropic medication**					
	**Controls (** ***n*** **= 64)**		**Prev. depr.** ^**b**^ **(** ***n*** **= 109)**				**Controls (** ***n*** **= 62)**		**Prev. depr. (** ***n*** **= 85)**			
	***M***	***SD***	***M***	***SD***	***t***	***p***	***M***	***SD***	***M***	***SD***	***t***	***p***
Age	37.1	12.3	37.5	11.3	−.17	.863	37.2	12.1	38.2	11.6	−.51	.611
Education (years)	16.7	2.5	16.3	2.3	1.11	.268	16.8	2.5	16.4	2.3	.88	.378
Vocabulary^c^ (T-score)	61.5	6.5	60.4	6.1	.98	.329	61.6	6.6	60.2	6.6	1.20	.231
Matrix (T-score)	58.5	5.5	57.5	6.3	.97	.333	58.5	5.6	57.6	6.2	.92	.358
BDI-II	4.3	5.2	10.9	7.9	−6.60	.000	4.0	4.8	10.1	7.9	−5.76	.000
BAI	3.2	4.1	5.6	5.0	−3.46	.001	2.9	3.8	5.6	4.9	−3.66	.000
Recurrent depr.			62%						60%			

^a^All participants were female.

^b^Prev. depr. = Previous depression.

^c^Some participants did not complete Vocabulary, primarily because it was not administered to those whose first language was not Norwegian. On a few occasions Vocabulary was skipped because the participant got too tired or because of time constraints. Of the 173 participants in this sample, 151 completed Vocabulary.

#### Executive functions

Essential outcome data on executive functions are presented in Table 2. In summary there were no significant differences between groups on any of the executive functions measures.

**Table 2:** **Executive functions**

	**Controls**		**Prev. depr.**		***t*** **-test**			
	***M***	***SD***	***M***	***SD***	***df***	***t***	***p***	***d***
CWI^a^ Inhibition	11.0	2.2	10.6	2.7	144	.82	.412	−.16
CWI Inhibition/switching	10.5	2.5	10.2	2.5	145	.79	.431	−.12
SSRT^b^ neutral	161	53	171	57	144	1.23	.262	.14
SSRT angry	168	48	175	54	144	.84	.405	.18
EPST Perseverative resp.^c^	20.9	14.7	18.6	15.2	137	1.12	.266	−.16

^a^CWI = Color-Word Interference, scaled score of completion time.

^b^SSRT = Stop-Signal Reaction Time, reported in milliseconds.

^c^EPST = Emotional Picture Sorting Task, Perseverative responses. *M*, *SD*, and Cohen’s *d* based on actual values, *t*-test was performed with Log transformed values.

##### Emotional picture sorting task

Two participants did not perform the EPST due to technical issues. Three participants were excluded from analyses because their failure to complete any categories may indicate that these participants did not understand the instructions or chose to not follow the instructions. Furthermore, three scores on the main outcome variable Perseverative responses were excluded as outliers, exceeding 2.5 standard deviations above the mean. Across the remaining 139 participants, the mean number of trials to complete the task was 106.2 (*SD* 21.0), the mean of categories completed was 5.0 (*SD* 1.5); this performance was not significantly different between groups (*t*s <1.4, *p*s > .1). The variable Perseverative responses was log transformed for analyses and was not significantly different between groups.

##### Stop signal task

One person did not complete the SST due to a hearing impairment that affected her perception of the stop signal. Across all remaining 146 participants the mean percent successful stop for the neutral and angry conditions were 52% and 51%, mean go reaction times were 438 ms and 443 ms, respectively. There were no differences between groups on these variables (all *t*s <1.2, all *p*s > .2). *t*-tests also showed no differences between groups on the main outcome variables SSRT neutral and SSRT angry (Table 2).

##### Color-word interference test

Time to complete task was converted to scaled scores based on available norms [37]. *t*-tests showed no differences between groups on CWI Inhibition and Inhibition/switching scaled scores.

Cognitive impairment has been reported to increase with number of depressive episodes [49,50]. We made a post hoc decision to repeat the *t*-tests excluding participants with a history of only one episode, thus comparing the never depressed participants to participants that had two or more previous episodes of depression (n = 51). This procedure did not influence the group means, *t*-values or *p*-values in any noticeable way.

#### Emotion regulation and rumination

Sum scores for the rumination factors reflection and brooding were calculated based on the factor items identified by W Treynor, R Gonzalez and S Nolen-Hoeksema [35], each factor score comprising five item scores. Sum scores for the emotion regulation strategies cognitive reappraisal and expressive suppression were calculated from six and four item scores, respectively [36]. *t*-tests confirmed statistically significant differences on all four variables, see Table 3. Previously depressed participants reported more reflection, brooding and expressive suppression, and less cognitive reappraisal.

**Table 3:** **Emotion regulation and rumination**

	**Controls**		**Prev. depr.**		***t*** **-test**			
	***M***	***SD***	***M***	***SD***	***df***	***t***	***p***	***d***
Cognitive reappraisal	30.6	6.5	28.1	6.8	171	−2.49	.014	−.37
Expressive suppression	11.1	3.9	12.9	4.8	171	2.37	.019	.39
Rumination full scale	35.5	8.9	53.6	11.7	165^a^	−10.63	.000	1.31
Brooding	8.0	2.6	11.6	3.5	162^b^	−7.80	.000	.99
Reflection	9.1	3.5	11.8	3.4	171	−5.07	.000	.74

^a^Six cases were excluded due to missing item values.

^b^One case was excluded due to missing item values. Equal variances not assumed.

A logistic regression analysis was performed for further investigation of the ability of ERQ and RRS variables to predict history of depression. The model containing cognitive reappraisal, expressive suppression, brooding and reflection as predictor variables and history of depression as outcome variable was statistically significant, χ2 (4, N = 173) = 56.90, p < .001, indicating that the model was able to predict which individuals had experienced clinical depression. The model explained between 28.2% (Cox & Snell R Square) and 38.4% (Nagelkerke R Square) of the variance in experienced depression, and correctly classified 75% of the cases. As shown in Table 4, brooding was the strongest predictor of previous depression, followed by reflection and expressive suppression. Reappraisal did not significantly contribute to the explanatory power of the logistic regression model.

**Table 4:** **Logistic regression: predicting likelihood of previous depression**

	**B**	**S.E.**	**Wald**	***df***	***p***	**Odds ratio**	**95% C.I. for Odds ratio**	
							**Lower**	**Upper**
Cogn. Reappraisal	−.02	.03	.24	1	.623	.98	.92	1.05
Exp. Suppression	.09	.05	3.92	1	.048	1.10	1.00	1.20
Brooding	.30	.07	18.53	1	.000	1.35	1.18	1.55
Reflection	.15	.06	6.14	1	.013	1.16	1.03	1.31
Constant	−4.53	1.48	9.40	1	.002	.01		

Our second objective was to investigate the relationships between executive functions and emotion regulation. Data from traditional and emotional EF tests were entered as predictors in linear regression models with cognitive reappraisal, brooding and reflection as outcome variables. As shown in Table 5 none of the executive functions models significantly predicted cognitive reappraisal, brooding or reflection.

**Table 5:** **Multiple regression analyses for executive functions and emotion regulation**

	**Predictors**	**Dependent**	***R*** ^***2***^	***df***	***F***	***p***
*Model 1*	CWI Inhibition	Cognitive reappraisal	.021	2,143	1.15	.223
	CWI Inhibition/switching					
*Model 2*	CWI Inhibition	Brooding	.031	2,143	2.32	.102
	CWI Inhibition/switching					
*Model 3*	CWI Inhibition	Reflection	.007	2,143	.50	.606
	CWI Inhibition/switching					
*Model 4*	Log Persev. responses	Cognitive reappraisal	.024	3,140	1.15	.332
	SSRT neutral					
	SSRT angry					
*Model 5*	Log Persev. responses	Brooding	.031	3,140	1.50	.217
	SSRT neutral					
	SSRT angry					
*Model 6*	Log Persev. responses	Reflection	.016	3,140	.77	.511
	SSRT neutral					
	SSRT angry					

## Discussion

The previously depressed participants matched never-depressed individuals on all neutral and emotional executive functions tasks. The previously depressed individuals reported that they more often respond to negative emotion with rumination and suppression and more rarely with reappraisal. A logistic regression model including all four factors of rumination and emotion regulation indicated that brooding, reflection, and suppression, but not reappraisal, predicted previous depression. The latter finding coincides with a proposition stating that the use of maladaptive strategies may be more important to psychopathology than the non-use of adaptive strategies [51].

The absence of differences in executive performance between groups was unexpected and calls for a closer inspection. All participants in the current study were carefully assessed for current and previous depression and most other common mental health disorders, and categorized according to history of depression. Marked differences on current depressive and anxiety symptoms as reflected by Beck scales is a further indication of the clinically different characteristics of the groups. We were able to match the groups and avoid potential confounds in age, education, and general cognitive abilities. For the analyses of executive functions we also excluded individuals who were taking psychotropic medication. Whereas executive function, as indicated by the color-word interference test, is only marginally above the general population mean in both our participant groups, education is high and estimated IQ is approximately one standard deviation above the population mean. Although this prevents generalization to subgroups with low education and IQ, it is a strength of this study that the patient and control groups are highly similar in education and general cognitive abilities. Many studies of cognitive correlates of depression include severely impaired inpatients who tend to have high comorbidity, including alcohol or drug abuse, somatic health problems, and lower education. Such comorbidities complicate interpretation of results. Our results indicate that, on group level, previously depressed participants with relatively high education and IQ, and low comorbidity, are unimpaired in both neutral and emotional EF tasks. An alternative interpretation is that our groups were different in executive function, but that the tasks used in this study were not sensitive to the differences. This is an unlikely explanation for the Stroop task, which has been shown to differentiate between euthymic MDD participants and controls [8]. In contrast, this explanation cannot be ruled out for the emotional EF tasks, which were new modifications of established EF paradigms. However, the effect sizes are similarly small for Stroop and the other executive tasks in our study. We therefore believe that the absence of significant differences between the groups on the executive tasks reflects the true state of our participants, at least in terms of non-emotional executive functioning. But the intensity of emotional stimuli in our tasks may have been too low to induce a significant effect. A comparison of stop-signal reaction time for the conditions neutral and angry suggests that the emotional effect was small.

Consistent with previous research we found more trait rumination among the previously depressed individuals [15,52]. Thus, correlational data suggests that both brooding and reflection may have negative effects on mood and depression risk. Reflection was initially described as an adaptive form of rumination [35], and this proposition gained some further support [53], although J Joormann, DE Nee, MG Berman, J Jonides and IH Gotlib [54] found that more reflection (but not brooding) was associated with working memory interference. Correlational data cannot rule out non-causal explanations, e.g. that reflection does not in itself confer depression risk but is an attempt to cope with the noxious effects of brooding. However, a meta-analysis indicates that both factors are related to symptoms of depression, although the association is stronger for brooding [55]. Whether reflection leads to increased depressive symptoms depends on the interaction with other coping strategies [56]. Importantly, in a prospective study, I Demeyer, E De Lissnyder, EH Koster and R De Raedt [57] found that impaired cognitive control for emotional information influenced depressive symptoms one year later, and that this was fully mediated by rumination.

Contrary to some other studies we found more suppression and less reappraisal in the previously depressed group, and inclusion criteria may explain the differences. Whereas T Ehring, B Tuschen-Caffier, J Schnülle, S Fischer and JJ Gross [18] included only participants whose BDI score was smaller than 10, and J Joormann and IH Gotlib [15] used specified criteria to ensure full remission, we included participants who were currently not depressed according to diagnostic criteria, regardless of their current BDI scores or sub-clinical symptoms. Depending on the research question, excluding participants with negative emotions from studies of emotion regulation may imply excluding an important part of the topic. Emotion regulation tendencies are relatively stable [58] whereas mood and symptoms of depression naturally vary with time within individuals. By definition the purpose of emotion regulation is to influence emotion, and our rationale for studying emotion regulation is that it may, over time, influence psychological well-being. Given that individual differences in emotion regulation makes some individuals more vulnerable to depression [12,15] it can be expected that differences in emotion regulation may lead to differences in symptoms as reflected by BDI scores. In this context, strict inclusion criteria based on BDI or similar symptom assessments may eliminate important natural variance in the phenomena that are studied.

Executive performance did not significantly predict cognitive reappraisal or rumination. The CWI scaled scores show that executive performance is slightly above the general population average in this sample. The absence of executive dysfunction may explain why executive function was unrelated to rumination and reappraisal, and does not exclude the possibility of such correlations in samples with executive dysfunction. Another possible explanation relates to the complexity of executive processes. Inhibition is not strictly a unitary construct: inhibition of external distractors, internal distractors, and prepotent responses are partially separable components [29,59]. Both Stop-Signal and Stroop have been classified as response inhibition tasks [29,59]. In our understanding the involvement of inhibitory subcomponents is rather uncertain for the Stroop task, which may also rely on inhibition of cognitively prepotent irrelevant information. Different aspects of inhibition are likely to contribute differentially to the control of rumination and reappraisal, and the observed correlations will depend on the choice of tasks. J Joormann and IH Gotlib [15] used a Negative Affective Priming tasks to show that reduced inhibition of negative material was associated with less use of reappraisal and more use of suppression in currently depressed, previously depressed, and never depressed participants. Reduced inhibition of negative material was associated with increased rumination only in currently depressed participants [15]. However, the use of negative priming to indicate inhibitory control is controversial.

The fact that we used non-verbal material in the emotional tasks is another possible explanation, as previous studies have indicated that rumination may be associated with performance in tasks using verbal [22], but not facial [26], stimuli in depressed participants. It is also a reasonable assumption that rumination is primarily a verbal process [26,54] and that it consequently should be closer related to performance in verbal, as opposed to non-verbal, tasks. On the other hand, the visual perception of facial expressions is deeply rooted in humans by evolution and not dependent on language or reading skills. Faces are also relevant in the current context because depression seems to be characterized by a disruption in the interpersonal domain [60]. Furthermore, executive functions are typically defined as general high-level control mechanisms that operate on various other processes [61,62]. In this perspective, the contribution from executive inhibitory mechanisms should be the same regardless of whether the task is presented with words or faces, and variance in performance between verbal and non-verbal tasks must be attributed to non-executive processes.

The majority of our previously depressed participants had received cognitively oriented psychotherapy, and we cannot rule out the possibility that this may have had some impact on our main outcome variables. Cognitive therapy for depression will typically attempt to promote antecedent-focused emotion regulation, including reappraisal, and reduce depressive rumination, and may possibly also change executive performance [63]. However, based on the observation that our previously depressed participants are clearly different from controls on rumination and emotion regulation we find it unlikely that initial executive impairments in at-risk individuals have been eliminated by psychotherapy in the current sample.

### Limitations and future directions

This study included only female participants. Participant gender has previously proven to not affect executive functions in remitted MDD compared to controls [8], so including male participants would most likely not influence this aspect of our results. By contrast, the observed group differences in rumination and emotion regulation cannot necessarily be generalized to male populations because men and women process emotional events differently [64]. Men and women use partially different strategies to cope with emotional distress in everyday life [65-67], and the relation between rumination and depression is stronger in women [55]. According to Thayer and colleagues, women rely more heavily on inhibitory processes for normal social and emotional functioning, and perseverative thinking and rumination may partly be caused by deficient inhibition [66]. Different aspects of inhibition and their relation to emotion regulation and rumination in men, both in remission from depression and in never-depressed controls, require attention in future studies.

The correlational nature of our data calls for cautious interpretation, but there is reason to trust the proposition that the differences in rumination and emotion regulation constitute vulnerability to depression in our sample. According to a meta-study, the relationship between rumination and depression is equivalent in longitudinal and cross-sectional data [55].

Stronger neural activation may in some instances compensate for the impaired performance otherwise associated with trait rumination. In an fMRI study of healthy, never-depressed individuals, M-A Vanderhasselt, S Kuhn and R De Raedt [68] found that brooding was associated with increased activation in right dorsolateral prefrontal cortex when successfully disengaging from negative material. This indicates that healthy high-brooders need more attentional control to disengage from negative information [68]. BCY Lo, S Lau, S-h Cheung and NB Allen [69] found increased late positive potential on medial scalp sites (Fz, Cz and Pz electrodes) among high ruminators when shifting between emotional material while in an induced sad mood state. An interesting continuation of the current research would be to use ERP data to investigate whether increased activation may explain why the previously depressed perform similar to control participants on executive tasks.

## Conclusions

The previously depressed participants did not exhibit impaired executive functions as compared to never depressed subjects, but they more often respond to negative emotion with rumination and suppression and more rarely with reappraisal. Trait rumination is not related to executive functions in this population, which indicates that rumination may be targeted directly in preventive interventions.

## Abbreviations

EF: Executive functions
AUDIT: Alcohol Use Disorder Identification Test
DUDIT: Drug Use Disorders Identification Test
CWI: Color-Word Interference test
ESST: Emotional Stop-Signal Task
EPST: Emotional Picture Sorting Task
ERQ: Emotion Regulation Questionnaire
RRS: Ruminative responses scale

## References

[1] American Psychiatric Association (2000). Diagnostic and Statistical Manual of Mental Disorders, 4th Edition, Text Revision.

[2] Kessler RC, Berglund P, Demler O, Jin R, Merikangas K, Walters EE (2005). Lifetime prevalence and age-of-onset distributions of DSM-IV disorders in the National Comorbidity Survey Replication. Arch Gen Psychiatry.

[3] Solomon DA, Keller MB, Leon AC, Mueller TI, Lavori PW, Shea M, Coryell W, Warshaw M, Turvey C, Maser JD, Endicott J (2000). Multiple recurrences of major depressive disorder. Am J Psychiatry.

[4] Boland RJ, Keller MB, Gotlib IH, Hammen CL (2009). Course and outcome of depression. Handbook of Depression.

[5] National Institute for Health and Clinical Excellence (2009). Depression in adults: the treatment and management of depression in adults.

[6] Snyder HR (2013). Major depressive disorder is associated with broad impairments on neuropsychological measures of executive function: a meta-analysis and review. Psychol Bull.

[7] Wagner S, Doering B, Helmreich I, Lieb K, Tadic A (2012). A meta-analysis of executive dysfunctions in unipolar major depressive disorder without psychotic symptoms and their changes during antidepressant treatment. Acta Psychiatr Scand.

[8] Bora E, Harrison BJ, Yücel M, Pantelis C (2013). Cognitive impairment in euthymic major depressive disorder: a meta-analysis. Psychol Med.

[9] Hasselbalch BJ, Knorr U, Kessing LV (2011). Cognitive impairment in the remitted state of unipolar depressive disorder: a systematic review. J Affect Disord.

[10] Bradley BP, Mogg K, Lee SC (1997). Attentional biases for negative information in induced and naturally occurring dysphoria. Behav Res Ther.

[11] Williams J, Mathews A, MacLeod C (1996). The emotional Stroop task and psychopathology. Psychol Bull.

[12] De Lissnyder E, Koster EH, Derakshan N, De Raedt R (2010). The association between depressive symptoms and executive control impairments in response to emotional and non-emotional information. Cogn Emot.

[13] Peckham AD, McHugh R, Otto MW (2010). A meta-analysis of the magnitude of biased attention in depression. Depress Anxiety.

[14] Vanderhasselt M-A, De Raedt R, Dillon DG, Dutra SJ, Brooks N, Pizzagalli DA (2012). Decreased cognitive control in response to negative information in patients with remitted depression: an event-related potential study. J Psychiatry Neurosci.

[15] Joormann J, Gotlib IH (2010). Emotion regulation in depression: relation to cognitive inhibition. Cogn Emot.

[16] Nolen-Hoeksema S (1991). Responses to depression and their effects on the duration of depressive episodes. J Abnorm Psychol.

[17] Ehring T, Fischer S, Schnülle J, Bosterling A, Tuschen-Caffier B (2008). Characteristics of emotion regulation in recovered depressed versus never depressed individuals. Person Indiv Diff.

[18] Ehring T, Tuschen-Caffier B, Schnülle J, Fischer S, Gross JJ (2010). Emotion regulation and vulnerability to depression: spontaneous versus instructed use of emotion suppression and reappraisal. Emotion.

[19] Holden C (2005). Sex and the suffering brain. Science.

[20] Nolen-Hoeksema S, Wisco BE, Lyubomirsky S (2008). Rethinking rumination. Perspect Psychol Sci.

[21] Jose PE, Brown I (2008). When does the gender difference in rumination begin? gender and age differences in the use of rumination by adolescents. J Youth Adolesc.

[22] Joormann J, Gotlib IH (2008). Updating the contents of working memory in depression: interference from irrelevant negative material. J Abnorm Psychol.

[23] Philippot P, Brutoux F (2008). Induced rumination dampens executive processes in dysphoric young adults. J Behav Ther Exp Psych.

[24] Watkins E, Brown R (2002). Rumination and executive function in depression: an experimental study. J Neurol Neurosurg Psychiatry.

[25] Whitmer AJ, Gotlib IH (2012). Switching and backward inhibition in major depressive disorder: the role of rumination. J Abnorm Psychol.

[26] Goeleven E, De Raedt R, Baert S, Koster EH (2006). Deficient inhibition of emotional information in depression. J Affect Disord.

[27] Harmer CJ, Goodwin GM, Cowen PJ (2009). Why do antidepressants take so long to work? a cognitive neuropsychological model of antidepressant drug action. British J Psychiatry.

[28] Bestgen Y, Dupont V (2000). Is negative priming a reliable measure for studying individual differences in inhibition?. Current Psychol Cogn.

[29] Friedman NP, Miyake A (2004). The relations among inhibition and interference control functions: a latent-variable analysis. J Exp Psychol-Gen.

[30] Tipper SP (2001). Does negative priming reflect inhibitory mechanisms? a review and integration of conflicting views. Quarterly J Exp Psychol A-Hum Exp Psychol.

[31] Beck AT, Steer RA, Brown GK (1996). Beck Depression Inventory-II (BDI-II) Manual [Norwegian translation, 2005].

[32] Beck AT, Steer RA (1990). Beck Anxiety Inventory Manual [Norwegian translation 2005].

[33] Babor TF, Higgins-Biddle JC, Saunders JB, Monteiro MG (2001). AUDIT - Alcohol Use Disorders Identification Test. Guidelines for use in primary care.

[34] Berman AH, Bergman H, Palmstierna T, Schlyter F (2005). Evaluation of the Drug Use Disorders Identification Test (DUDIT) in criminal justice and detoxification settings and in a Swedish population sample. Eur Addict Res.

[35] Treynor W, Gonzalez R, Nolen-Hoeksema S (2003). Rumination reconsidered: a psychometric analysis. Cogn Ther Res.

[36] Gross JJ, John OP (2003). Individual differences in two emotion regulation processes: implications for affect, relationships, and well-being. J Pers Soc Psychol.

[37] Delis DC, Kaplan E, Kramer J (2001). Delis-Kaplan Executive Function System.

[38] Stroop J (1935). Studies of interference in serial verbal reactions. J Exp Psychol.

[39] Aker M, Landrø NI: **Executive control of emotional processing: a set-shifting task**. *Clin Neuropsychol,* in press 10.1080/13854046.2014.984762 25422031

[40] Grant DA, Berg E (1948). A behavioral analysis of degree of reinforcement and ease of shifting to new responses in a Weigl-type card-sorting problem. J Exp Psychol.

[41] Heaton RK (2005). Wisconsin Card Sorting Test Computer Version 4.

[42] Lundqvist D, Flykt A, Öhman A: *The Karolinska Directed Emotional Faces - KDEF.* CD ROM from Department of Clinical Neuroscience, Psychology section, Karolinska institutet, ISBN 91-630-7164-9; 1998. http://emotionlab.se/resources/kdef.

[43] Logan GD, Cowan WB, Davis KA (1984). On the ability to inhibit simple and choice reaction time responses: a model and a method. J Exp Psychol-Hum Percept Perform.

[44] Osman A, Kornblum S, Meyer DE (1986). The point of no return in choice reaction time: controlled and ballistic stages of response preparation. J Exp Psychol-Hum Percept Perform.

[45] Verbruggen F, Chambers CD, Logan GD (2013). Fictitious inhibitory differences: how skewness and slowing distort the estimation of stopping latencies. Psychol Sci.

[46] Verbruggen F, De Houwer J (2007). Do emotional stimuli interfere with response inhibition? evidence from the stop signal paradigm. Cogn Emot.

[47] Wechsler D (1999). Weschsler Abbreviated Scale of Intelligence (WASI). (Norwegian version: 2007).

[48] Clarke H, Walker S, Dalley J, Robbins T, Roberts A (2007). Cognitive inflexibility after prefrontal serotonin depletion is behaviorally and neurochemically specific. Cereb Cortex.

[49] Kessing LV (1998). Cognitive impairment in the euthymic phase of affective disorder. Psychol Med.

[50] Paelecke-Habermann Y, Pohl J, Leplow B (2005). Attention and executive functions in remitted major depression patients. J Affect Disord.

[51] Aldao A, Nolen-Hoeksema S (2010). Specificity of cognitive emotion regulation strategies: a transdiagnostic examination. Behav Res Ther.

[52] Thomas E, Elliott R, McKie S, Arnone D, Downey D, Juhasz G, Deakin J, Anderson I (2011). Interaction between a history of depression and rumination on neural response to emotional faces. Psychol Med.

[53] Joormann J, Dkane M, Gotlib IH (2006). Adaptive and maladaptive components of rumination? diagnostic specificity and relation to depressive biases. Behav Ther.

[54] Joormann J, Nee DE, Berman MG, Jonides J, Gotlib IH (2010). Interference resolution in major depression. Cogn Aff Behav Neurosci.

[55] Olatunji BO, Naragon-Gainey K, Wolitzky-Taylor KB (2013). Specificity of rumination in anxiety and depression: a multimodal meta-analysis. Clin Psychol-Sci Pr.

[56] Marroquin BM, Fontes M, Scilletta A, Miranda R (2010). Ruminative subtypes and coping responses: active and passive pathways to depressive symptoms. Cogn Emot.

[57] Demeyer I, De Lissnyder E, Koster EH, De Raedt R (2012). Rumination mediates the relationship between impaired cognitive control for emotional information and depressive symptoms: a prospective study in remitted depressed adults. Behav Res Ther.

[58] John OP, Gross JJ (2004). Healthy and unhealthy emotion regulation: personality processes, individual differences, and life span development. J Personality.

[59] Nigg JT (2000). On inhibition/disinhibition in developmental psychopathology: views from cognitive and personality psychology and a working inhibition taxonomy. Psychol Bull.

[60] Gotlib IH, Hammen CL (2002). Handbook of Depression.

[61] Miyake A, Friedman NP, Emerson MJ, Witzki AH, Howerter A (2000). The unity and diversity of executive functions and their contributions to complex “frontal lobe” tasks: a latent variable analysis. Cogn Psychol.

[62] Alvarez JA, Emory E (2006). Executive function and the frontal lobes: a meta-analytic review. Neuropsychol Rev.

[63] Alexopoulos GS, Raue P, Arean P (2003). Problem-solving therapy versus supportive therapy in geriatric major depression with executive dysfunction. Am J Geriatr Psychiatry.

[64] Dolcos F, Iordan AD, Dolcos S (2011). Neural correlates of emotion–cognition interactions: a review of evidence from brain imaging investigations. J Cogn Psychol.

[65] Matud M (2004). Gender differences in stress and coping styles. Person Indiv Diff.

[66] Thayer JF, Rossy LA, Ruiz-Padial E, Johnsen BH (2003). Gender differences in the relationship between emotional regulation and depressive symptoms. Cogn Ther Res.

[67] Garnefski N, Teerds J, Kraaij V, Legerstee J, van den Kommer T (2004). Cognitive emotion regulation strategies and depressive symptoms: differences between males and females. Person Indiv Diff.

[68] Vanderhasselt M-A, Kuhn S, De Raedt R (2011). Healthy brooders employ more attentional resources when disengaging from the negative: an event-related fMRI study. Cogn Aff Beh Neurosci.

[69] Lo BCY, Lau S, S-h C, Allen NB (2012). The impact of rumination on internal attention switching. Cogn Emot.

